# Oxidative stress and the cardiovascular effects of air pollution

**DOI:** 10.1016/j.freeradbiomed.2020.01.004

**Published:** 2020-05-01

**Authors:** Mark R. Miller

**Affiliations:** University/BHF Centre for Cardiovascular Science, University of Edinburgh, 47 Little France Crescent, Edinburgh, EH4 3RL, United Kingdom

**Keywords:** Air pollution, Cardiovascular, Diesel exhaust, Particulate matter, Oxidative stress

## Abstract

Cardiovascular causes have been estimated to be responsible for more than two thirds of the considerable mortality attributed to air pollution. There is now a substantial body of research demonstrating that exposure to air pollution has many detrimental effects throughout the cardiovascular system. Multiple biological mechanisms are responsible, however, oxidative stress is a prominent observation at many levels of the cardiovascular impairment induced by pollutant exposure. This review provides an overview of the evidence that oxidative stress is a key pathway for the different cardiovascular actions of air pollution.

## Highlights

• This review provides a detailed summary of the evidence for a role of oxidative stress in the cardiovascular effects of air pollution.
• Evidence from epidemiological studies, controlled exposures in human subjects, **in vivo** animal models and **in vitro** assays is assessed.
• Evidence is discussed in regards to different facets of cardiovascular (patho)physiology e.g. vascular tone, arrhythmia, thrombosis, stroke.
• Emphasis is given to the action of particulates in air pollution, especially combustion-derived particles from vehicle exhaust.
• The evidence suggests that oxidative stress plays a contributing role in the cardiovascular dysfunction caused by exposure to air pollution.

## Graphical abstract

**Figure:**
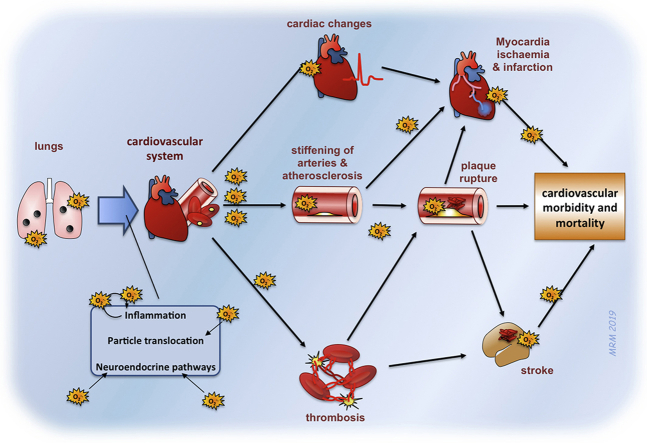

## Abbreviations

AHA: American Heart Association
AhR: aryl hydrocarbon receptors
BBB: blood brain barrier
BC: black carbon
CAPs: concentrated ambient particles
CIMT: carotid intima media thickness
CO: carbon monoxide
CO_2_: carbon dioxide
DE: diesel exhaust
DEP: diesel exhaust particulate
ECG: electrocardiogram
ET-1: endothelin-1
HDL: high density lipoprotein
HRV: heart rate variability
ICAM-1: intercellular adhesion molecule-1
LDL: low density lipoprotein
LOX-1: lectin-like oxidized low density lipoprotein receptor
NAC: N-acetylcysteine
NADPH oxidase: nicotinamide adenine dinucleotide phosphate oxidase
NMDA: N-methyl-d-aspartate
NO: nitric oxide
NO_2_: nitrogen dioxide
NOS: nitric oxide synthase
O_3_: ozone
8-OH-dG: 8-oxo-2′-deoxyguanosine
oxLDL: oxidized low density lipoprotein
PAHs: poly-aromatic hydrocarbons
PM: particulate matter
PM_10_: particulate matter with a diameter <10 μm
PM_2.5_: particulate matter with a diameter <2.5 μm
SO_2_: sulphur dioxide
SOD: superoxide dismutase
VCAM-1: vascular cell adhesion molecule-1
vWF: von Willebrand factor

## 1 Introduction

Air pollution has far-reaching consequences on biodiversity, and its impact on human health has moved this issue to the top ranks of the political agenda at an international level. Air pollution is the number one environmental risk factor for mortality and the fifth greatest risk factor for all-cause mortality, overall [1]. Recently, it has been revealed that air pollution has effects throughout the body [2,3], however, its effects on the cardiovascular system, in particular, carry a disproportionate burden both in terms of morbidity and mortality. Indeed, due to high prevalence of cardiovascular disease globally and the intrinsic fatality of many cardiovascular conditions, more than two thirds of the mortality attributed to air pollution arise from cardiovascular causes, in particular ischaemic heart disease and cerebrovascular disease [1,4]. Additionally, cardiovascular complications are likely to play important contributing roles to the action of air pollution in the progression of diseases in other organs [5,6].

Great progress has been made in establishing the mechanisms underlying the cardiovascular effects of air pollution. It has become clear that oxidative stress plays a key role in the cardiovascular effects of many air pollutants. This topic was reviewed in detail in 2012 [7], with the 2010–2016 data expertly reviewed in Kelly & Fussell 2017 [8]. This review is an amalgamation of previous evidence, together with more recent data (January 2016 to October 2019). By structuring the evidence into different facets of the cardiovascular pathophysiology, the intention is that this review will provide a comprehensive overview of role of oxidative stress in the cardiovascular effects of air pollution.

## 2 Air pollution

Air pollution includes a vast range of substances derived from many different sources and chemical reactions within the atmosphere. A brief overview is given here, and readers are referred to accompanying papers in this special issue for further details. Airborne pollutants can arise from both natural sources (e.g. forest fires, volcanic eruptions, aerosolised soil and dusts, pollen and moulds) and anthropogenic sources (e.g. industry, power plants, traffic, household heating, cooking, construction, mechanical wear, agriculture, etc.). ‘Ambient’ air pollution refers to outdoor air pollution, and has received the greatest attention historically, although indoor sources of air pollution are gaining traction, especially in terms of the burden of disease in developing nations. The bulk of research into ambient air pollution tends to centre on urban air pollution due to, among other reasons, the high density of urban populations, greater levels of traffic-derived emissions and increasing urbanisation of societies worldwide.

Urban pollution is a complex cocktail of chemicals. Gases such as sulphur dioxide (SO_2_), carbon dioxide (CO_2_), carbon monoxide (CO), ozone (O_3_) and nitrogen dioxide (NO_2_) are present in varying amounts. Gaseous pollutants have the potential to cause short- and long-term health effects, possibly in an additive manner to particulates [9]. Many of these gases have oxidative properties, and induction of oxidative stress (together with inflammation) is a likely mechanism by which they can affect human health [10]. Semi-volatile species such as benzene, naphthalene, formaldehyde, polyaromatic hydrocarbons (PAHs) exist as liquid droplets, but can also transition between gaseous and particulate phases of air pollution [11]. Additionally, there are numerous sources of minute airborne particulate matter (PM). PM is categorised according to particle size. Coarse particles (PM_10_) are particles with a diameter of 10 μm or less, fine particles (PM_2.5_) have a diameter of 2.5 μm or less, and ultrafine particles (or “nanoparticles”) have a diameter of 100 nm or less (although precise definitions of these categories can be distinct in different disciplines). PM is monitored and regulated in the environment through stationary monitoring networks that measure PM_10_ and (slightly less frequently) PM_2.5_. It is not practically possible to widely measure ultrafine PM using monitoring networks in the environment at present. Elemental and organic carbon form a significant part of urban PM (especially that derived from combustion) but non-carbon constituents such as various mineral dusts, sea salt, ammonium, nitrates, sulfates, and others, are also present [12]. The composition of particles is one of the key physiochemical properties determining the biological response to inhaled PM. Organic carbon species (PAHs, nitro-PAHs, alkanes, alkenes, alkyl-benzenes, quinones, etc.) and redox-active transition metals are frequently implicated in the health effects of urban PM, and the availability of these chemicals on the surface of PM influences the biological response to these particles [[13], [14], [15], [16], [17], [18]].

Particle size is also an important factor for the health effects of PM. Size is a determinant of the degree of penetration into the lung and ability (or inability) of biological clearance mechanisms to remove inhaled PM [19,20]. Importantly, the size also has an immediate effect on the relative surface area of the PM, with small particles having a significantly greater surface area than the equivalent mass of a larger particle of the same material (although this is complicated somewhat by particle agglomeration in the air and in biological fluids). Accordingly, health associations are often greater and/or more consistent for PM_2.5_ than PM_10_ [9,21]. There is a general assumption that ultrafine particles could pose a greater risk to health due to their larger relative reactive surface area, and their ability to penetrate deep into the alveoli of the lungs and into the bloodstream [22,23]. Vehicle exhaust is a prominent sources of ultrafine particles in urban PM, and diesel exhaust (DE) is of particular interest due to the greater proportion of ultrafine PM compared with gasoline/petrol engine emissions, as well as the tendency to be associated with high levels of co-pollutants such as NO_2_ [24,25].

Many of the above pollutants and their constituents have some capacity to induce health effects. However, in relation to the cardiovascular system, epidemiological associations tend to be more consistent for the particulate components [9,26]. For this reason, this review will largely focus on the cardiovascular actions of PM and especially that of vehicle-derived emissions as prominent sources of ultrafine PM.

## 3 Air pollution and cardiovascular disease: overview of epidemiological evidence

**Fig. 1:**
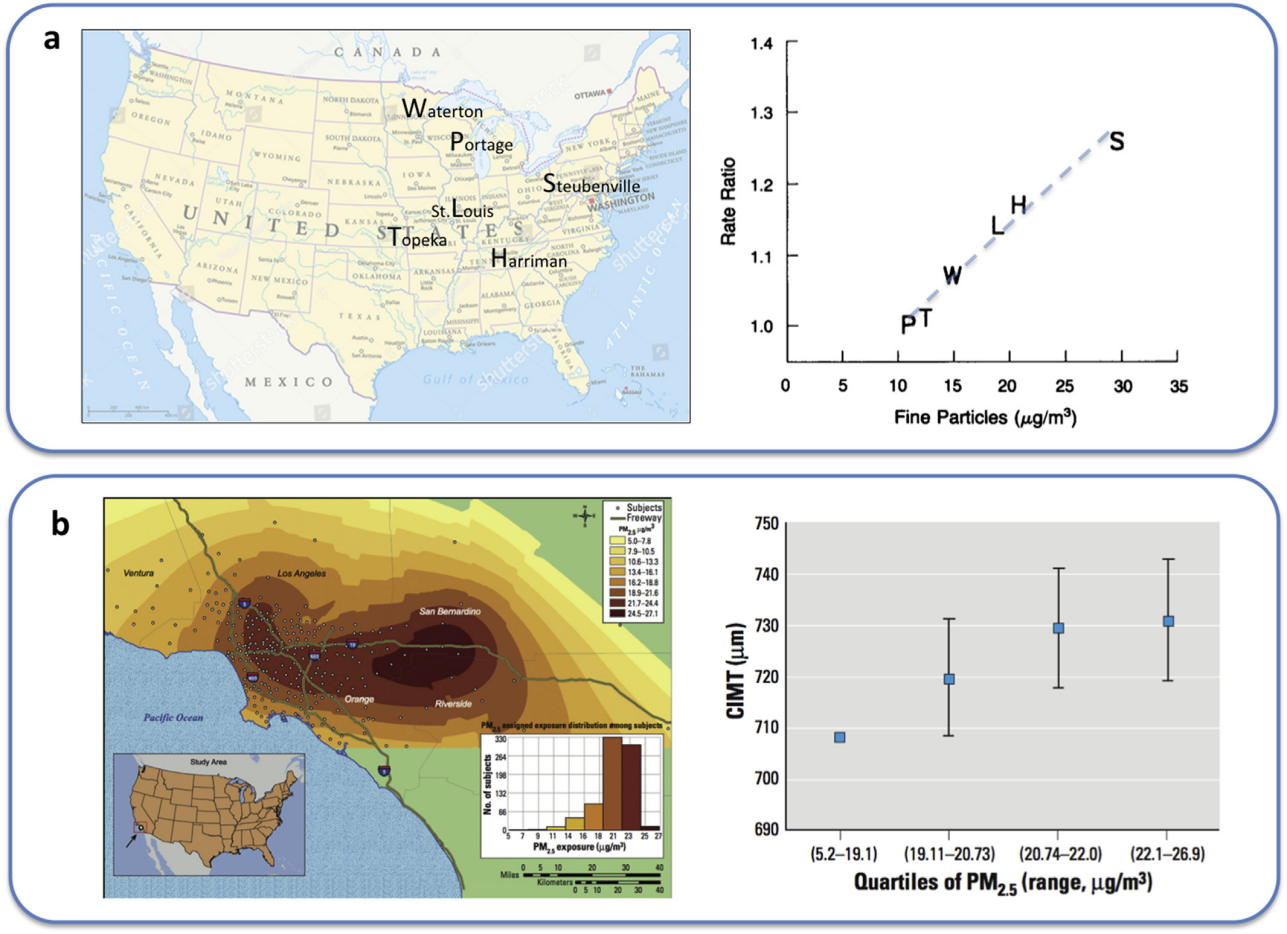
Association between fine particulate matter (PM_2.5_) and: a) risk of mortality and b) carotid atherosclerosis. a) Letters represent first letter of city marked on map of USA. Dotted line is approximate correlation added as indicator of the linearity of the relationship. Data from Dockery et al. 1993 [27]; b) Map shows modelled PM_2.5_ air pollution in area surrounding Los Angeles. CIMT = carotid intima media thickness. Data from Kunzli et al. 2005 [30].

The cardiovascular effects of air pollution came to prominence in the early 1990s. In 1993, Dockery and colleagues examined the relationship between PM_2.5_ and hospital admissions/deaths from cardiovascular disease in six North American cities with broadly similar demographics [27]. They found a strikingly linear relationship between levels of PM_2.5_ and cardiovascular morbidity and mortality (Fig. 1a). An adjusted mortality-rate ratio of 1.37 (95%CI: 1.11–1.68) was found for cardiopulmonary mortality between the least (11 μg/m^3^ PM_2.5_) and most (30 μg/m^3^ PM_2.5_) polluted cities. A later study by the group [28] expanded this data to include 51 metropolitan areas throughout the USA. It was estimated that a decrease of 10 μg/m^3^ PM_2.5_ would increase average life expectancy by approximately 7 months. Furthermore, a 2007 study of more than 65,000 women in the USA demonstrated a 24% increase in risk of a cardiovascular event and an alarming 76% increase in the risk of death from cardiovascular disease [29]. Lastly, a seminal study by Kunzli et al. [30] showed a relationship between exposure to PM_2.5_ across Los Angeles, USA, and atherosclerosis (a chronic disease of the vasculature that underlies coronary artery disease and many other cardiovascular conditions). A difference of 10 μg/m^3^ PM_2.5_ was associated with a 4–6% increase in carotid-intima thickness (CIMT); a measure of atherosclerosis in the arteries of the neck that is predictive of disease in other vascular beds (Fig. 1b).

Air pollution is associated with mortality and cardiovascular events (e.g. a heart attack or a stroke) in both the long- and short-term. Utilising data from three large US cohorts (359–500 thousand participants), Pope et al. demonstrated that long-term exposure to air pollution (1–4 years) was associated with an 8–18% increase in cardiovascular mortality per 30 μg/m^3^ PM_2.5_ [31]. An 8 year follow-up of ~5000 individuals found almost a doubling of the risk of cardiovascular mortality in individuals living in close proximity to a major road [32]. In the short-term, increases in cardiovascular mortality [33,34] and coronary events [35] have been associated with ambient PM on the same and preceding day. The coronary effects of traffic-derived pollution may occur even earlier, as individuals presenting with myocardial infarction were more likely to have been in traffic 1–2 h beforehand [[36], [37], [38]].

The dose-response relationship between PM and cardiovascular mortality still requires investigation, especially at low and high doses. Current evidence suggests that there is a linear relationship between moderate levels of PM and mortality, followed by a plateau in mortality rates at higher levels (“supralinear”) [[39], [40], [41]]. Importantly, relatively low levels of air pollution can promote cardiovascular disease long-term [40]. Notably, these associations hold true for levels of PM_2.5_ that are currently below international guidelines (e.g. World Health Organisation; annual PM_2.5_ <10 μg/m^3^) [42,43].

The volume of epidemiological evidence stretches far beyond mortality and broad metrics of morbidity. Indeed, air pollution has been shown to be associated with most cardiovascular conditions, including coronary artery disease [29,44,45], cardiac arrhythmia and arrest [35,46,47], acute myocardial infarction [45,48,49], heart failure [48,[50], [51], [52]], cerebrovascular disease [29,[53], [54], [55], [56], [57]], peripheral arterial disease [58,59] and venous thromboembolism [60,61]. Comprehensive reviews of the mechanistic evidence for these associations have concluded that there is a strong case for causality between air pollution and a wide range of cardiovascular endpoints [62]. This conclusion is concisely summarised in the seminal American Heart Associations (AHA) systematic reviews in 2004 and 2010 [9,63]: “evidence is consistent with a causal relationship between PM_2.5_ exposure and cardiovascular morbidity and mortality” [9]. The weight of evidence has only been bolstered in the prevailing years. A 2015 expert position paper [64] concluded that “There is now abundant evidence that air pollution contributes to the risk of cardiovascular disease and associated mortality, underpinned by credible evidence of multiple mechanisms that may drive this association”, stressing that “Air pollution should be viewed as one of several major modifiable risk factors in the prevention and management of cardiovascular disease”.

## 4 Oxidative stress in the cardiovascular effects of air pollution

Oxidative stress has been cemented as a key pathway underlying the cardiovascular effects of air pollution [7,65,66]. Both epidemiological studies and controlled exposure studies in human subjects have provided strong evidence for oxidative pathways and these foundations have been built upon by a network of mechanistic studies in animals and cellular models (expertly summarised in Ref. [8]). The 2010 AHA statement concluded that “At a molecular level, oxidative stress as a critically important cause and consequence of PM-mediated cardiovascular effects has a sound experimental basis” [9]. This review aims to be a comprehensive review of the role of oxidative stress in the cardiovascular effects of air pollution (for further information on the wider effects of PM-induced oxidative stress see Refs. [[66], [67], [68]]). Studies have been identified that contain both an association between air pollution and cardiovascular function, and mechanistic evidence for oxidative stress (e.g. assessment of the oxidative potential of a pollutant, measurement of a biomarker of oxidative stress, identification of a source of free radicals, exploration of genetic polymorphisms conferring altered susceptibility to oxidative stress, or prevention/reversal with antioxidant compounds). The sheer volume of literature on this topic prohibits detailed critical analysis of individual assays, study designs and inconsistences. Nonetheless, all evidence in this overview is derived from peer-reviewed journals and expert book chapters. Furthermore, by structuring the review by different facets of cardiovascular system, it is clear that oxidative stress is a common, and important, mechanism in many of the different processes linking air pollution to cardiovascular mortality.

### 4.1 Vascular tone/endothelial dysfunction

The tone of blood vessels is crucial to the distribution of blood around the body, and plays a key role in both the maintenance of homeostasis and the response to stimuli. Endothelial cells are the thin layer of cells that line the inner surface of blood vessels. The endothelium acts as an interface between the blood and vascular wall, both physically and through the synthesis and release of a range of active mediators. Endothelial dysfunction plays a key early role in vascular impairment and disease. Subsequently, the excessive contractility and loss of vasodilator responses caused by endothelial dysfunction are hallmarks of chronic cardiovascular disease.

**Fig. 2:**
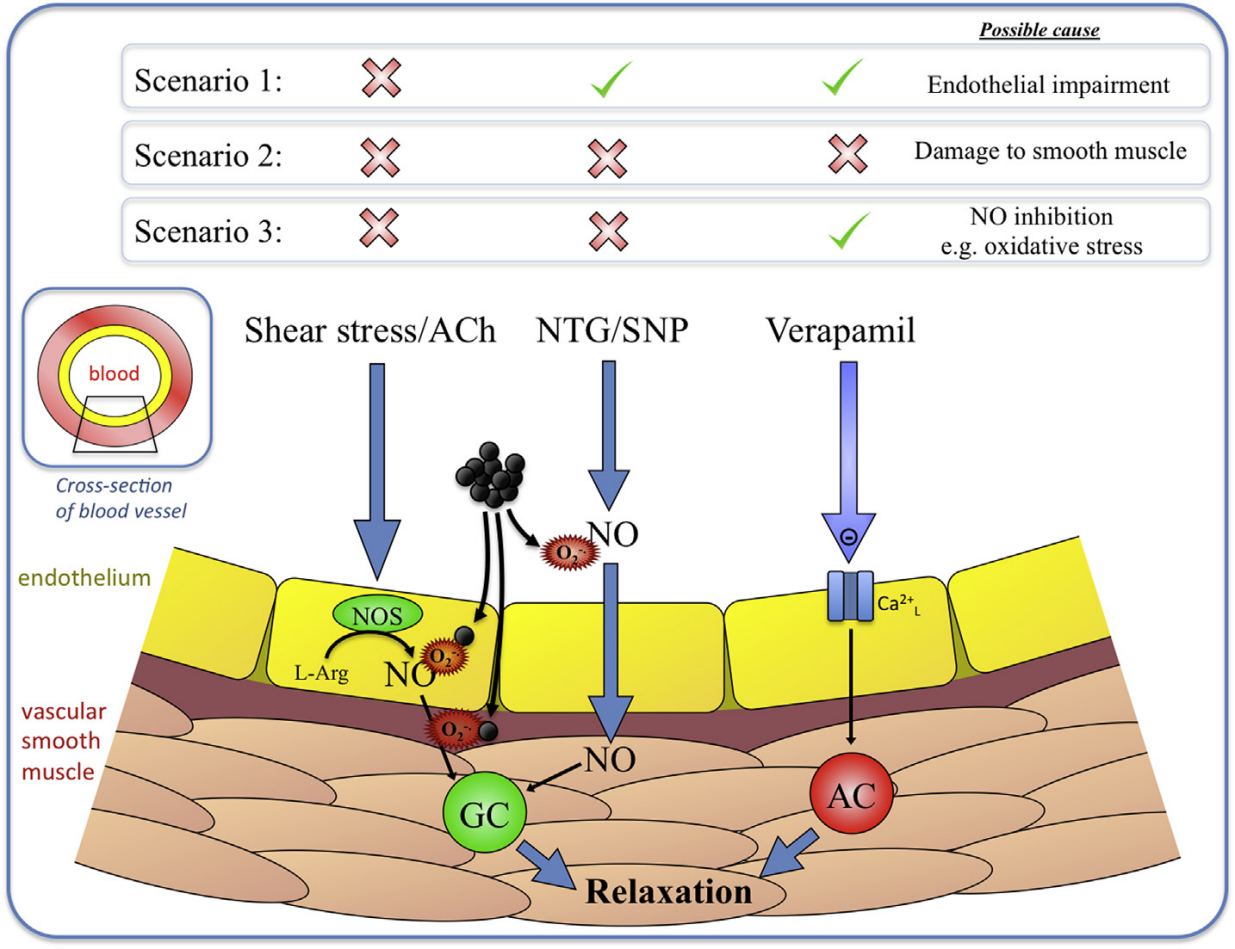
Exposure to PM inhibits vasodilatation mediated through endothelial and nitric-oxide pathways. A combination of different stimuli and drug infusions can be used to explore the mechanisms by which vasodilatation is impaired (see example scenarios). Flow-mediated dilatation (FMD) and infusion of drugs such as acetylcholine (ACh) and bradykinin (BK) stimulate endothelial cells to synthesise nitric oxide (NO). NO diffuses to the smooth muscle to activate guanylate cyclase (GC) which ultimately induces relaxation of the vascular smooth muscle and dilatation of the blood vessel. Drugs such as nitroglycerin (NTG or glyceryl trinitrate) and sodium nitroprusside (SNP) act independently of the endothelium to generate NO from their molecular structure. Drugs such as verapamil (and isoprenaline in rodent models) activate receptors on vascular smooth muscle cells to induce vasodilatation independently of NO. Exposure to PM tends to inhibit pathways involving NO, but not NO-independent pathways. This pattern suggests that oxidative stress is a prominent mechanism of action, due to the scavenging of NO by superoxide free radicals (O_2_^-.^). Other abbreviations: AC = adenylate cyclase, Ca^2+^_L_, L-type calcium channel NOS = nitric oxide synthase.

**Epidemiology.** In 2005 O'Neill et al. demonstrated that PM_2.5_ exposure was associated with a decreased in vasodilator response in the brachial artery of diabetic individuals [69]. Subsequent evidence implicated oxidative stress as a potential mechanism. The reduction in brachial artery diameter to PM_2.5_ and black carbon (BC; used as an indicator for combustion-derived PM) in elderly individuals was associated with increased in plasma endothelin-1 (ET-1; a potent vasoconstrictor molecule) and oxidative stress (thiobarbituric reactive substances) [70]. Similar observations were made for the reduction in microvascular function in middle-aged and elderly individuals, that demonstrated that smaller arteries (which play a substantial role in blood pressure regulation, with high blood pressure ("hypertension") being a prominent risk factor of cardiovascular disease) were also narrowed by PM [71]. Accordingly, PM exposure has been regularly associated with small, but significant, elevations in blood pressure [[72], [73], [74], [75], [76], [77]]. The hypertensive effect of air pollution is likely to be multifactorial in terms of both the contributing biological mechanisms and different air pollutants [72]. A role for oxidative stress has been postulated by the nature of the pattern of vascular impairment induced by PM (Fig. 2). The oxygen free radical superoxide scavenges nitric oxide (NO), a key endothelial-cell derived mediator that controls vasodilatation of blood vessels. PM_2.5_ exposure was associated with impairment of both flow-mediated dilatation (endothelium-dependent) and vasodilation to nitroglycerin (an endothelium-independent NO-releasing vasodilator). This pattern of impairment (inhibition of vasodilators acting through NO) is suggestive of oxidative stress [69]. A role for both oxidative stress and inflammation is supported by observations that the attenuation of flow-mediated dilatation in response to PM_2.5_ was greater in individuals with glutathione-S-transferase M1 polymorphisms, and associated with increases in myeloperoxidase [78]. Similarly, healthy volunteers taken on a 1.5-h drive along a busy roadway showed diminished reactive hyperemia responses (a measure of endothelial-mediated vasodilatation) and reduced levels of plasma nitrite (a surrogate for endothelial NO) [79]. This effect was not apparent when cabin filters were used to lower particulate levels in the vehicle.

Various blood and urine markers of oxidative stress are upregulated in participants with greater levels of PM exposure (e.g. Refs. [[80], [81], [82], [83], [84], [85], [86], [87], [88], [89], [90], [91], [92]]). Additionally, personal monitoring of pollutants has been used to show that cumulative indoor and outdoor exposure to ultrafine particles is associated with oxidation of DNA in the blood [93]. Traffic-related pollutants, specifically, appear to closely linked to circulating biomarkers of oxidative stress [87,89]. Exposure to PM decreased endothelial-mediated dilatation in the microvasculature of elderly individuals in Los Angeles, USA [94]. The degree of impairment was greatest for PM with characteristics of traffic-derived PM (black carbon and NO_2_) and was correlated with the oxidative potential of the PM *in vitro*.

There is evidence that polymorphisms in antioxidant enzymes lead to a greater hypertensive response to pollutant exposure in comparison to those with the standard genetic alleles [95]. In contrast, other studies have not found the associations between PM and vascular function/blood pressure to be accompanied by oxidative stress biomarkers [96,97] or genetic status of antioxidant enzymes [98].

There is a growing body of evidence linking air pollution to metabolic syndrome and diabetes; diseases that are frequently associated with cardiovascular complications, including microvascular dysfunction [99,100]. While the associations between air pollution and diabetes are not discussed in detail in the current review, it is worthwhile noting that increases in both insulin resistance and biomarkers of oxidative stress were associated with air pollution exposure in children [101]. Finally, ultrafine particles altered levels of endothelial progenitor cells (that are likely to be involved in the repair of endothelial damage) and leukocyte free radical production in non-smokers in Copenhagen [102].

**Controlled Exposure Studies.** Controlled exposure studies in healthy volunteers have been vital in exploring the biological mechanisms of specific pollutants without many of the confounding variables of epidemiological studies. Controlled exposures to dilute diesel exhaust (DE) induced a compensatory increase in levels of antioxidants to the lung lining fluid as a response to the oxidative insult to the lungs [103,104], setting the scene for use of this approach to study the cardiovascular effects of pollutants. A 2-h inhalation of concentrated ambient particles (CAPs; ~150 μg PM/m^3^) and ozone (120 ppb) from the city of Toronto induced vasoconstriction of brachial arteries [105]. A potential involvement of a systemic oxidative response was suggested by the greater levels of oxidation of the DNA of blood monocyte following controlled exposure to roadside ultrafine PM [106]. A program of work by led by Newby and Mills utilised controlled exposures to identify the mechanisms by which exposure to DE impaired cardiovascular function. Their first study demonstrated that a 1-h exposure of DE (~300 μg PM/m^3^) caused a potent attenuation of vasodilator responses in the forearm [107]. By utilising a series of different vasodilator infusions, they demonstrated that DE caused inhibition of endothelium-dependent vasodilatation (to acetycholine, bradykinin) and endothelium-independent NO donor drugs (to sodium nitroprusside), but not vasodilators that act independently of the NO pathway (verapamil) (see Fig. 2). This pattern of inhibition strongly supports a role for oxidative stress, as opposed to another means of vascular dysfunction such as non-specific damage to endothelial cells or de-regulation of vascular smooth muscle contractility (although other mechanisms such as down-regulation of the NO target enzyme, guanylate cyclase, could also be involved). This profile of vascular impairment was consistently observed in subsequent studies by these researchers [[108], [109], [110]]. Inhalation of DE altered both antioxidant genes and genes regulating vascular homeostasis (e.g. vascular endothelial growth factor) in peripheral blood monocytes [111]. Loss of endothelial NO-signalling contributed to the increased sensitivity to circulating ET-1 following acute exposure to DE [112]. Controlled exposure to DE also impaired skin microvascular responses to endothelium-dependent vasodilators, but not an exogenous NO donor drug [113]. Although the response to the NO donor drug was not altered, blood serum from volunteers induced the release of superoxide in cultured endothelial cells, the extent of which was related to the total dose of the volunteer received.

Supplementation with antioxidant-rich fish oils have been found to limit the extent of endothelial dysfunction in response to controlled exposure to CAPs [114]. Conversely, another study found that N-acetylcysteine (NAC), a compound with antioxidant properties (albeit it can also affect cell signalling through means unrelated for oxidative stress), promoted vasoconstriction to DE rather than prevented it [115]. The reasons for this unexpected finding were not immediately apparent although could involve changes in vasoconstrictive eicosanoids or alterations in expression of basal antioxidants. The particulate components seem to drive the acute vascular effects of DE [110,116], and not gases such as NO_2_ [117] or O_3_ [118]. In this regard, a recent study found that controlled exposure to higher concentrations of ozone increased blood levels ET-1, but actually decreased blood levels of nitrotyrosine (a marker of the reaction product of NO and superoxide) [119].

**Animal models.** Rodent models have been beneficial in exploring the mechanisms underlying the increased vasoconstriction/impaired vasodilation. The mechanistic evidence gathered by these studies provides strong evidence that oxidative stress may play a significant contributing role in mediating the impairments [7,120]. Inhalation of DE in rats led to impaired endothelium-dependent relaxation in coronary arteries, an effect that could be reversed with superoxide scavengers [121]. Two weeks exposure of rats to PM_2.5_ (albeit at high concentrations of 600 μg/m^3^) impaired pulmonary artery vasodilator function and decreased eNOS expression, in line with a compensatory increase in vascular superoxide dismutase (SOD; an enzyme which converts superoxide into oxygen and hydrogen peroxide) [122]. Interestingly, nitric oxide synthase (NOS; the enzymatic source of NO) inhibitors, or additional of NOS co-factors, prevented the impairment, suggesting that NOS uncoupling (causing generation of superoxide from NOS instead of NO) was an important mechanism. In support of this, sub-chronic exposure to PM_2.5_ led to depletion of the co-factors needed for the NO-generation from endothelial NOS [123]. Other cellular sources of oxidative stress have been implicated. A role for nicotinamide adenine dinucleotide phosphate oxidase (NADPH oxidase) has been implicated in vascular impairment and elevated blood pressure induced by PM_2.5_ [[123], [124], [125]] and diesel exhaust particulate (DEP) [126,127]. Exposure of rats to residual oil-fly ash caused rapid attenuation of relaxation responses in the microvasculature and adherence of myeloperoxidase-containing leukocytes to the vessel wall, suggesting that leukocytes enzymatically contributed to free radicals linked to the endothelial dysfunction [128].

Oxidative stress also appears to play a role in the effects of PM exposure on circulating endothelial progenitor cells [129]. Indeed, Haberzettl and colleagues demonstrated that CAPs inhalation in mice led to a plethora of mechanistic links between endothelial progenitor cells, vascular insulin resistance, inflammation and oxidative stress, building on the evidence that air pollution is associated with both cardiovascular disease and metabolic disease [130]. The same group demonstrated that nine days inhalation of CAPs depleted circulating endothelial progenitor cells and led to an impaired angiogenic response (the growth of new blood vessels) in a mouse model of hind limb ischaemia [131]. Mice overexpressing extracellular superoxide dismutase in the lung were protected against this effect, suggesting pulmonary oxidative stress has downstream consequences on the vasculature.

***In vitro* experiments.** Similarly to *in vivo* studies, isolated blood vessels directly treated with DEP also exhibit impaired endothelial-dependent vasodilatation and NO-mediated vasodilation, but not relaxations caused by NO-independent vasodilators [132]. These findings provide support for the contention that if these particles reach the systemic circulation (by translocation from the lung into the pulmonary blood vessels) they could directly impair vascular function through oxidative stress without the need for prior interaction with the lung or inflammatory cells [132]. Scavengers of oxygen free radicals and inhibitors of enzymatic sources of free radicals can prevent the direct vascular impairment induced by DEP (see Ref. [7]). Albeit, the direct effect of PM on endothelial cells may be modest in comparison to that produced when the particles first interact with inflammatory cells [133].

Direct exposure of isolated brain capillaries to DEP increased oxidative stress and inflammation; findings that may have implications for blood brain barrier integrity following inhalation of pollutants [134]. Direct treatment of cultured endothelial cells with PM, DEP or motorcycle exhaust particles has also been shown to induce oxidative stress, alter endothelial cell signalling, upregulate adhesion molecules, down-regulate endothelial NOS and, ultimately, promote apoptosis [[135], [136], [137], [138], [139], [140], [141]]. Finally, NAC has been shown to attenuate several effects of PM (e.g. inflammation and downregulation of NOS) in endothelial cells [135,136,139,141,142].

### 4.2 Atherosclerosis

Endothelial dysfunction is an early initiating event in the vascular disease atherosclerosis. Loss of endothelial function and expression of adhesion molecules attracts and tethers circulating inflammatory cells to the vascular wall. Additionally, loss of NO and changes to endothelial cell phenotype encourage the oxidation of circulating lipids (e.g. low density lipoprotein (LDL) to oxidized LDL (oxLDL)) that are preferentially retained by inflammatory cells that begin to penetrate the damaged endothelial layer. The accumulation of both of inflammatory cells and lipids induces the formation of a fatty plaque in major arteries that grow into the lumen to impede blood flow. Erosion or rupture of advanced plaques is the trigger for thrombosis (a blood clot) that may occlude arteries causing a cardiovascular event such as a heart attack or stroke.

**Epidemiology.** Individuals with greater exposure to PM (e.g. based on pollution monitoring data close to residential address, or distance of the residence from a major road) exhibit greater degrees of atherosclerosis, as assessed by a number of methods such as arterial wall thickness, coronary calcification (a marker of advanced plaques) and reduction of lumen diameter in the retinal microvasculature (which can be used as a non-invasive indicator of early atherosclerosis with prognostic value for cardiovascular outcomes) [30,[143], [144], [145], [146]]. Exposure to ambient PM or BC has been associated with greater levels of inflammatory biomarkers and reduced antioxidant activity in the blood of elderly individuals with coronary artery disease [86]. The narrowing of retinal blood vessels was associated with PM exposure, paralleled by increases in circulating levels of micro-RNA implicated with oxidative stress [147]. Associations have been observed for CIMT and the oxidative capacity of PM_10_ collected in the year preceding the CIMT measurements [148].

Occupational exposure to vehicle emissions (e.g. bus drivers and garagemen) led to greater levels of several markers of systemic oxidative stress in comparison to comparative controls [83,149]. These included urinary 8-oxo-2′-deoxyguanosine (8-OH-dG; a marker of oxidative modification of DNA) and 15-isoprostanes, blood levels of protein carbonyls and nitrotyrosine, and lower levels of antioxidants in plasma. These observations were correlated to a number of pollutants, including PM_10_, PM_2.5_ and PAHs. There was no striking relationship with blood levels of LDL or high density lipoprotein (HDL) [83], although the study did not measure oxLDL specifically. Nonetheless, a similar study found greater levels of oxLDL and decreased levels of antioxidants in the blood of taxi drivers [150]. Furthermore, exposure to traffic-related air pollution in Shanghai, China, was associated with elevated levels of LDL. The effects on LDL were accompanied by increased blood pressure, indicators of insulin resistance and decreased antioxidant capacity [151]. An interesting study by Wu et al. recruited students in Beijing before and after moving to a university campus with higher pollution levels [152]. Increased exposure to PM_2.5_, especially PM rich in metals, led to higher oxLDL in the blood.

The adhesion molecules, vascular cell adhesion molecule-1 (VCAM-1) and intercellular adhesion molecule-1 (ICAM-1) are involved in the attraction and tethering of leukocytes to the blood vessel wall, thus have a role in both early and on-going atherosclerosis. Exposure to PM_2.5_ increased levels of soluble VCAM-1 and ICAM-1 in the blood, an effect that was greater in individuals that are null for glutathione-S-transferase M1 [153]. Antioxidant-rich fish oils have been shown to reduce levels of the circulating vasoconstrictor ET-1 that are associated with PM_2.5_ exposure [154]. Finally, extracellular vesicles carrying microRNA represent an emerging mechanism by which the pro-oxidative and pro-atherosclerotic effect of PM could be disseminated around the body [155].

**Controlled exposures in humans.** The acute nature of controlled exposure studies in human subjects does not lend itself to studying the chronic development of atherosclerosis. However, it should be borne in mind that controlled exposure studies have been shown to have acute cardiovascular effects that will promote the development of vascular disease (see other sections of this review). Two controlled exposure studies were identified that specifically implicate oxidative stress in the atherosclerotic effects of air pollution. Controlled exposure of healthy subjects to DE increased plasma-soluble lectin-like oxidized low density lipoprotein receptor (LOX-1) levels, which would be expected to mediate, at least in part, the accumulation of lipids within the vascular wall [156]. High density lipoprotein (HDL) acts to inhibit the oxidation of LDL, thus having a preventative action on the development of atherosclerosis. Controlled exposure to PM_2.5_, but not ozone, was shown to reduce the antioxidant activity of HDL in individuals with lower baseline HDL activity [157].

**Animal and *in vitro* models.** Due to the long-term development of atherosclerosis in humans (over decades) and limited means to non-invasively measure atherosclerosis clinically, the availability of rodent models of atherosclerosis has been a boon for studying the mechanisms underlying atherosclerosis. The apolipoliprotein-E knockout and LDL receptor knockout mice have been especially useful in this regard, as advanced atherosclerotic plaques can develop in these mice in a few weeks if they are fed a high-cholesterol diet. A number of studies have now shown that exposure to PM or vehicle exhaust particles accelerate the development of atherosclerosis in these mice, both in terms of increasing plaque size and promoting plaque vulnerability to rupture (reviewed in Refs. [7,120]). Oxidative stress has been implicated as a key mechanism for the pro-atherosclerotic effects of pollutants.

**Fig. 3:**
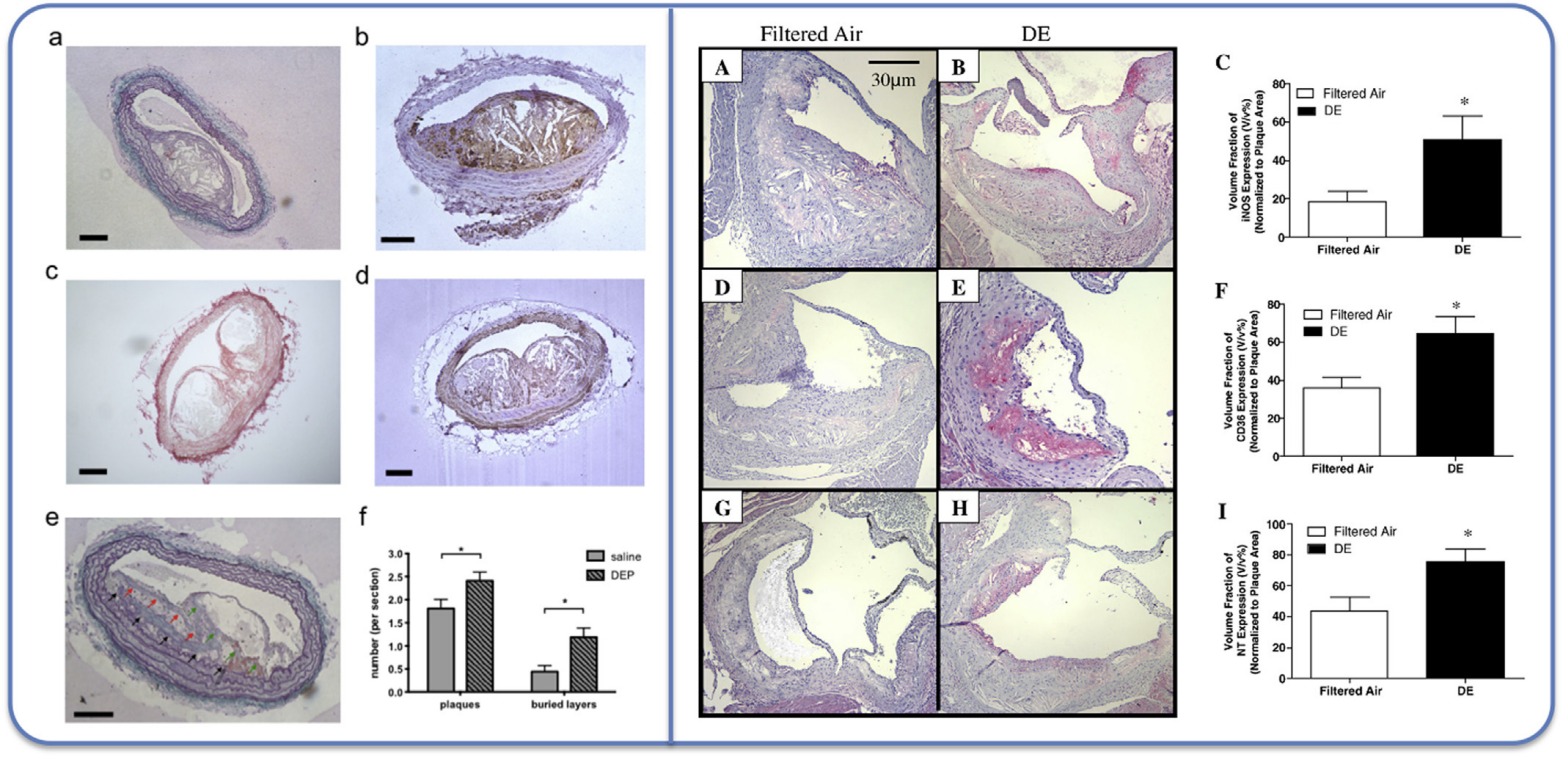
Different histochemical approaches to assess constituents of atherosclerotic plaques. *Left panel:* Plaques in the brachiocephalic artery of ApoE knockout mice instilled with diesel exhaust particles or control (saline) [161]. a) Cavities from lipids and cholesterol crystals, b) macrophage-derived foam cells (brown stain), c) collagen (red), d) smooth muscle cells (brown), e & f) buried fibrous layers (arrows). *Right panel:* Plaques in the aortic root of ApoE knockout mice inhaling diesel exhaust or control (filtered air). Data from Bai et al. 2011 [170]. A-C) Inducible nitric oxide synthase expression (pink/red), D-F) CD-3 expressing cells (cells derived from inflammatory cells; pink/red), G-I) nitrotyrosine staining as a marker of oxidative stress (pink/red).

PM and DE has been shown to oxidise LDL *in vitro* [158] and *in vivo* [159]. Furthermore, DEP exhibited synergistic effects with lipids on gene expression in cultured endothelial cells; an effect that was replicated by inhalation of ambient ultrafine PM in hypercholesterolaemic mice [160]. PM-exposed atherosclerotic mice exhibited biomarkers of systemic oxidative stress in blood and urine, as well as alterations in antioxidant systems [43,127,[160], [161], [162], [163], [164], [165], [166]]. Inhalation of vehicle exhaust increased levels of LOX-1 [156,167] and dysfunctional HDL [164,168]. The antioxidant and inflammatory pathways, nuclear factor erythroid-2 (Nrf-2) and aryl hydrocarbon receptor (AhR), played an interacting role in the atherosclerotic effects of air pollution [169]. Furthermore, the plaques from PM-exposed mice have greater levels of markers of oxidative stress, demonstrated, for example, by nitrotyrosine staining [170] (Fig. 3). Eight weeks inhalation of PM increased the thickness of coronary arteries in healthy rats, accompanied by upregulation of angiotensin pathways and decreases in hemogenase-1 levels [171]. The role of gaseous versus particulate components of vehicle exhaust may have different effects on markers of oxidative stress and plaque composition [172,173].

No research was found investigating the ability of antioxidant compounds to prevent the pro-atherosclerotic effects of pollutants. However, the antioxidant properties of selenium supplementation, was found to reduce both oxidative stress and VCAM-1 levels in healthy rats exposed to PM, which would be expected to influence the early stages of atherosclerosis [174].

### 4.3 Arrhythmia and heart rate variability

Heart rate variability (HRV) is a set of parameters derived from detailed analysis of heart rhythm from electrocardiogram (ECG) recordings. These parameters are indicative of the modulation of the electrical activity of the heart, in particular, its regulation by the autonomic nervous system. For most HRV parameters, a reduction is would confer a greater risk of developing cardiovascular conditions (at a population level). The availability of low-cost non-invasive monitors to assess HRV has been readily employed to address the cardiac effects of air pollution, increasingly so in panel studies simultaneously measuring personal air pollution in real-time.

**Epidemiology.** Although there is inconsistency between parameters and studies, overall, exposure to PM is associated with reduction in HRV (see Refs. [62,175]). In students in Taipei, Taiwan, both PM_10_ and PM_2.5_ were associated with decreases in several HRV parameters, as well as increases in blood 8-OH-dG and C-reactive protein [80]. Personal monitoring devices were also used to show associations between PM_2.5_ and HRV indices in Boston, USA, the effects of which were linked to urinary levels of 8-OH-dG [176]. A series of US studies by Schwartz and colleagues demonstrated associations between PM_2.5_/traffic exposure and several HRV parameters [[177], [178], [179], [180], [181]]. In many cases, alterations in HRV associated with PM_2.5_ were modified by genetic differences in antioxidant systems. Similar genetic susceptibility in antioxidant genes influenced associations between PM_2.5_ and corrected Q-T interval [95,182]. In the interests of balance, others have failed to demonstrate clear links between air pollutants, HRV and oxidative biomarkers [96,183].

Two groups have explored HRV during interventions to reduce exposure to air pollutants. Langrish et al. demonstrated that wearing an efficient facemask beneficially altered selected HRV parameters in patients with ischaemic heart disease walking alongside city-centre roads in Beijing, in comparison to performing the same walk without a mask [184]. Laboratory analysis showed that the Beijing PM had a substantial capacity to generate superoxide free radicals. Also in Beijing, Lee and colleagues made measurements of HRV in residents during the 2008 Olympic Games when stringent measures were taken to reduce air pollution [185]. While reduction of air pollution was shown to reduce levels of urinary markers of oxidative stress, HRV changes were inconclusive. The lack of clarity, however, could be explained by pollutant-specific effects as, while PM was reduced considerably, there were higher levels of ozone during this time. Dietary antioxidant supplementation, omega-3 fish oils in particular, was shown to limit the effects of air pollution on HRV in elderly individuals [186]. Reductions in oxidative stress were also partially to account for the beneficial effects of statins in preventing the effects of PM_2.5_ on high-frequency HRV parameters [177,187].

**Controlled exposures in humans.** A role for oxidative stress in the action of PM on HRV has been supported by controlled exposure studies. Acute exposure to ultrafine CAPs altered HRV and cardiac repolarization in patients with metabolic syndrome who were null for the glutathione S-transferase M1 allele, but not a comparative group from the general population [188]. Controlled exposure to street air in Copenhagen was associated with reductions in HRV, although there were no consistent changes in various oxidative biomarkers [189]. In contrast, Tong et al. found that exposure to CAPs reduced HRV in healthy volunteers, and this could be prevented by 4 weeks of oral omega-3 fatty acid supplements [187].

**Animal models.** Several studies have addressed the cardiac effects of pollutants in rodents, demonstrating that PM can alter HRV via autonomic imbalance and alterations in baroreceptor sensitivity [190]. Additionally, infusion of isoprenaline (isoproterenol) was used to promote arrhythmias, in rodent models [[191], [192], [193]]. Five hours inhalation of CAPs in rats led to changes in ECG that were accompanied by increase in oxidative stress in cardiac tissue [194] (although it should be noted that the assays used - chemiluminescence, thiobarbituric acid reactive substances – can also be increased by alteration in intermediary metabolism rather than oxidative stress). These effects could be prevented by inhibition of the renin-angiotensin system or pulmonary sensory receptors. The complex interplay between the oxidative stress and the autonomic system is highlighted by a study that demonstrated HRV changes to urban PM can be prevented by NAC, while the cardiac oxidative stress induced by the PM can be inhibited by blockade of the autonomic nervous system with beta-blockers [195]. Pulmonary instillation of DEP to rats, prior to coronary artery ligation, increased the incidence of cardiac arrhythmia and the extent of myocardial infarction to the ischaemia induced by ligation [196]. In this model, DEP-induced ischaemia induced greater levels of superoxide radical generation in the perfused heart. PM_2.5_ exposure to rats also induced changes in HRV and increased cardiac malondialdehyde, the effects of which were modified by co-exposure to ozone [197]. Lastly, the prolongation of cardiac action potential and arrhythmias induced by pulmonary exposure to DEP were prevented by NAC [198].

### 4.4 Myocardial ischaemia and infarction

Atherosclerosis of the coronary vasculature is the basis for ischaemic heart disease, where the restriction of blood flow leads to regions of cardiac ischemic that can manifest as angina. Occlusion of major coronary arteries can lead to prolonged ischaemia, resulting in death of the downstream myocardial cells resulting in myocardial infarction; fibrosis or death of regions of the heart leading to loss of cardiac function and obstruction to cardiac electrical conductance.

**Epidemiology.** Exposure to traffic has been shown to increase the incidence of acute myocardial infarction in the subsequent hours [[36], [37], [38]]. Additionally, hospital presentations/admissions for myocardial infarction were greater for those dwelling in areas of high air pollution [45,48,49]. Long-term exacerbation of atherosclerosis and rupture of plaques will make a substantial contribution to the associations between myocardial infarction and exposure to air pollution. However, it is likely that many different cardiovascular effects of air pollution will act in concert to increase the susceptibility to cardiovascular events [199].

Oxidative stress (raised levels of blood malondiadehyde) was found in patients with acute coronary events linked to black carbon exposure [200]. Pekkanen et al. measured S-T segment depression (a region of an electrocardiogram representative of ischaemic stress in coronary arteries) in patients with stable coronary artery disease while exercising in the city of Helsinki, Finland [201]. NO_2_, CO, PM_2.5_, particle number (used as a surrogate for ultrafine PM), but not coarse PM, were associated with an increased prevalence of S-T segment depression, with a lag of 2 days. The associations were stronger in those not taking beta-blockers, indicating that these effects may be more reflective of autonomic regulation of the heart rather than the coronary arteries, although oxidative stress was not specifically investigated as a mechanism in this study. Susceptibility to coronary events (cardiac mortality, non-fatal myocardial infarction, admission for unstable angina) has also been associated with black carbon exposure (used as a proxy for combustion-derived particles), with greater levels of malondialdehyde on admission to hospital [200]. Furthermore, the oxidative potential of PM_2.5_ (and, to some degree, ozone co-exposure) related to the risk of myocardial infarction [202]: particulates with the greatest oxidative potential were associated with ~8% increase in hospital admissions. NO_2_ exposure has been found to be associated with increased risk of acute myocardial infarction [203]. The risk varied with polymorphisms of genes involved in glutathione pathways, although the investigators were not able to identify statistically significant changes in precise genes.

**Fig. 4:**
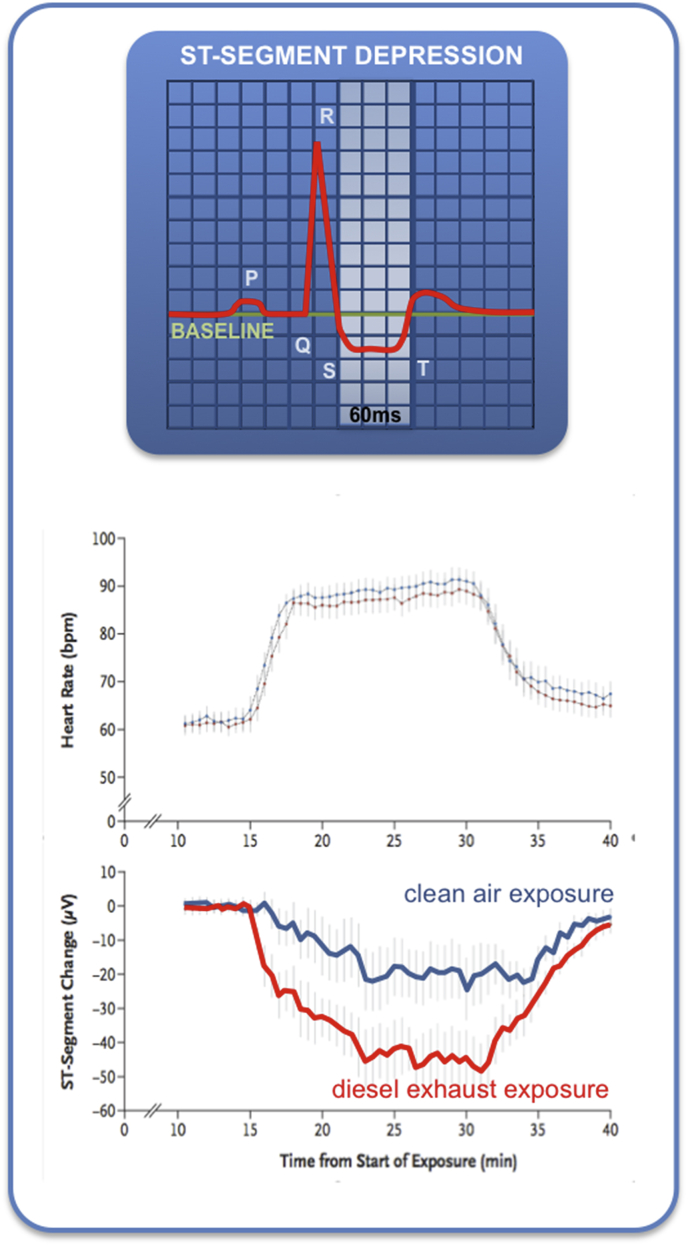
Acute exposure to dilute diesel exhaust exacerbates cardiac ischaemic stress in patients with ischaemic heart disease. Volunteers were asked to inhale diesel exhaust (300 μg/m^3^ for 1 h) during an exercise test. Ischaemic stress was measured as the extent of S-T segment depression in the ECG. Data from Mills et al. 2007 [204].

**Controlled exposures in humans.** The greater sensitivity of the heart to ischaemic stress was demonstrated using controlled exposures to diesel exhaust. A 2 h exposure to 300 μg/m^3^ DE in patients with ischemic heart caused a two-fold increase in the extent of S-T segment depression induced in an exercise test [204] (Fig. 4). This observation built on other work from this group showing that acute exposure to DE impaired vascular function through oxidative mechanisms [107].

**Animal models.** Ligation of coronary arteries in rodents can be used to induce myocardial infarction to address the mechanisms responsible. Rats instilled with DEP prior to ligation were more prone to the resultant arrhythmia and likelihood of sudden death [196]. Isolation of the heart afterward showed that the area of infarction was more than twice as large in rats exposed to DE than those that were non-exposed. Perfusion of the coronary blood vessel with a superoxide-sensitive spin-trap reagent demonstrated that there was an increase in free radical formation in the injured hearts of DE exposed mice. The antioxidant vanillic acid reduced the effects of PM_10_ on cardiac antioxidant levels, and partially improved mitochondrial disturbances after ischaemia reperfusion injury [205]. Similarly, selenium supplementation decreased the oxidative action of PM_2.5_ in cardiac tissue in rats [174]. Interestingly, 4 weeks inhalation of DE in healthy rats led to a broadly similar profile of cardiac gene expression (one linked with mitochondrial-derived oxidative stress) in healthy rats to that of air-exposed spontaneously hypertensive rats, suggesting DE induced similar cardiac changes to hypertension [206]. Gaseous pollutants can also exert similar effects, with a 1–2 month exposure to ozone increasing ischaemia-reperfusion injury in rats that was characterised by increases in cardiac lipid peroxidation and decreased SOD activity [207]. CO exacerbated myocardial injury and depletion of antioxidants in the heart of a rat model of myocardial infarction [208]. The CO levels used were at the upper ranges of ambient levels in heavily polluted urban cities (30 ppm), spiked with peaks representative of that very close proximity to vehicle exhaust (100 ppm).

***In vitro* experiments**. Direct addition of DEP to cultured cardiomyocytes reduced contractile function, an effect that could be partially prevented by antioxidants [209]. Similarly, PM_2.5_ that was known to reduce antioxidant capacity in mice, also caused decreased contractility of cardiomyocytes on direct exposure [210]. Finally, the direct cytotoxicity or apoptotic effect of various PM in cardiomyocytes could be inhibited by compounds with antioxidant properties (e.g. NAC or diemethylthiourea, a scavenger of hydroxyl radicals and hydrogen peroxide) [198,211]. It should be noted that, due to the reaction kinetics of free radicals with antioxidants in comparison to that of other target molecules, the physiological relevance of the use of antioxidants as free radical scavengers in *in vitro* assays has been questioned [212].

### 4.5 Cardiac remodelling and heart failure

Heart failure is the term used to describe the loss of the capacity of the heart as a result of long-term stress from coronary artery disease, prolonged high blood pressure, increased sympathetic drive, severe alterations in heart rhythm and damage to the heart from myocardial ischaemia, infarction or congenital defects and viral damage. It is characterised, ultimately, by a substantial loss of cardiac function so that the heart cannot deliver blood to meet the needs of the body. Hypertrophy (a remodelling and thickening of the cardiac wall) is common, whereby the heart attempts to compensate for the burden it is placed under, usually leading to a reduction in the efficiency of cardiac contractility and further pressure on the coronary arteries.

**Epidemiology.** Exposure to air pollution has been associated with an increased incidence of heart failure [48]. A meta-analysis [52] of global data found that a number of air pollutants (PM_10_, PM_2.5_, SO_2_, NO_2_, CO, but not O_3_) were associated with an increase in relative risk for heart failure. PM_2.5_was associated with a 2.12% increase in risk per 10 μg/m^3^, and showed the greater persistence in terms of lag effect - the only pollutant to be associated with a significant effect 2 days later (longer lags were not investigated). Left ventricular mass (i.e. hypertrophy) was been found to be greater in those residing closer to major roads [213,214]. These effects were magnified in those with polymorphisms for genes linked to inflammation and oxidative stress. Lastly, oxidative capacity of PM_2.5_ (via the dithiothreitol assay) showed stronger associations with hospital admissions for heart failure than PM_2.5_ (mass) alone [215] (although it should be noted the dithiothreitol assay is imprecise and has generated conflicting results elsewhere [216]).

**Fig. 5:**
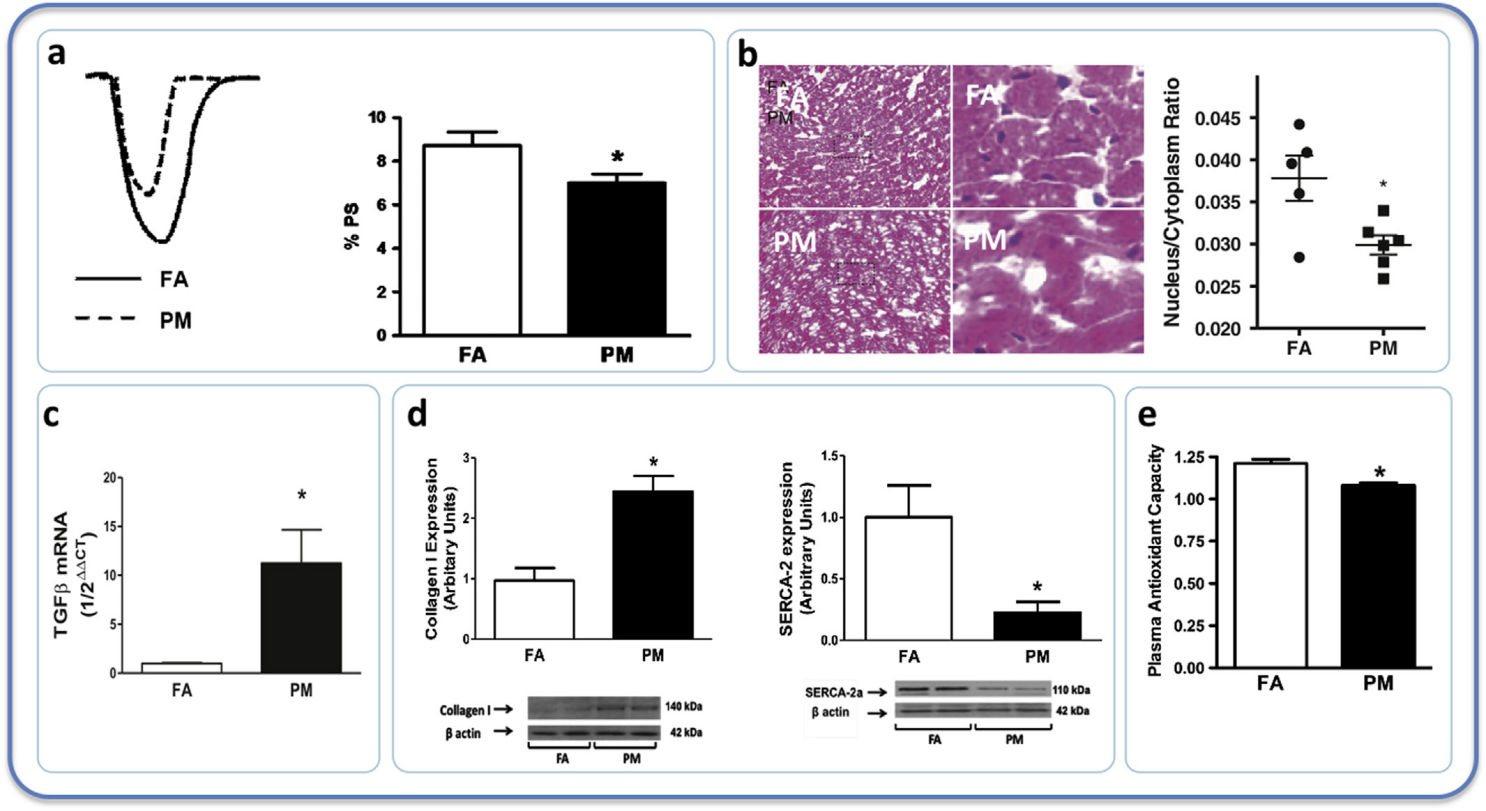
Nine months inhalation of PM in mice impairs cardiac contractility and promotes cardiac remodelling that is characteristic of heart failure. In cardiomyocytes PM exposure led to: a) reduction in contraction (PS: peak shortening), b) reduction in cell nucleus:cytoplasm ratio, c) increased expression of transforming growth factor (TGFB), d) increased protein expression of collagen and decreased SERCA-2 (a Ca^2+^-ATPase), e) reduction in plasma antioxidant capacity. Data from Wold et al. 2012 [210].

**Animal models.** PM_2.5_ exposure is associated with loss of cardiac contractility and increased collagen deposition in healthy rats [217]. This study also demonstrated that the same exposure in rats with myocardial infarction produced a compensatory increase in glutathione in the cardiac tissue. PM_2.5_ induced pulmonary inflammation and oxidative stress alongside right ventricular hypertrophy in a mouse model of heart failure [218]. An impressively intricate study by Wold et al. investigated the long-term effects of PM in a mouse model of heart failure, and cultured cardiomyocytes [210]. Nine months exposure to PM_2.5_ (moderate levels of CAPs: ~80 μg/m^3^) led to an increase in blood pressure of 10 mmHg that was linked to a decreased ventricular contractility and remodelling, without an overall change in coronary flow reserve. Cardiomyocytes transitioned towards a fibrotic phenotype rather than contractile (Fig. 5), and these effects were paralleled by a decrease in plasma total antioxidant capacity. Although the value of the plasma total antioxidant capacity assay is debated, these observations could suggest that oxidative stress was involved in the processes of myocyte transition. PM_2.5_ exacerbated the effects of angiotensin-induced hypertension, leading to cardiac hypertrophy [219]. These effects were mediated by RhoA/Rho-kinase signalling pathways, which the same group had previously shown to be involved in the hypertensive effects of PM_2.5_ via oxidative mechanisms [123]. Wang et al. found that inhalation of PM_2.5_ induced left-ventricular dysfunction in mice. In this experiment, AMP-activated protein kinase (a protein involved in the sensing and regulation of cellular metabolism) was implicated in both the pro-fibrotic response and generation of oxidative stress [220]. Left ventricular function has also been shown to be modified by 1–2 months ozone exposure; effects that were associated with lipid peroxidation in the heart, decreases in SOD activity and increases in cytokines [221].

### 4.6 Thombosis

Blood clotting is needed to prevent excessive bleeding on injury. However, coagulation needs to be finely regulated, as excessive clotting increases the risk of cardiovascular conditions caused by thrombotic occlusion of arteries, e.g. at sites of atherosclerotic plaque rupture/erosion or through lodging of emboli (blood-borne thrombus) in smaller downstream arteries.

**Fig. 6:**
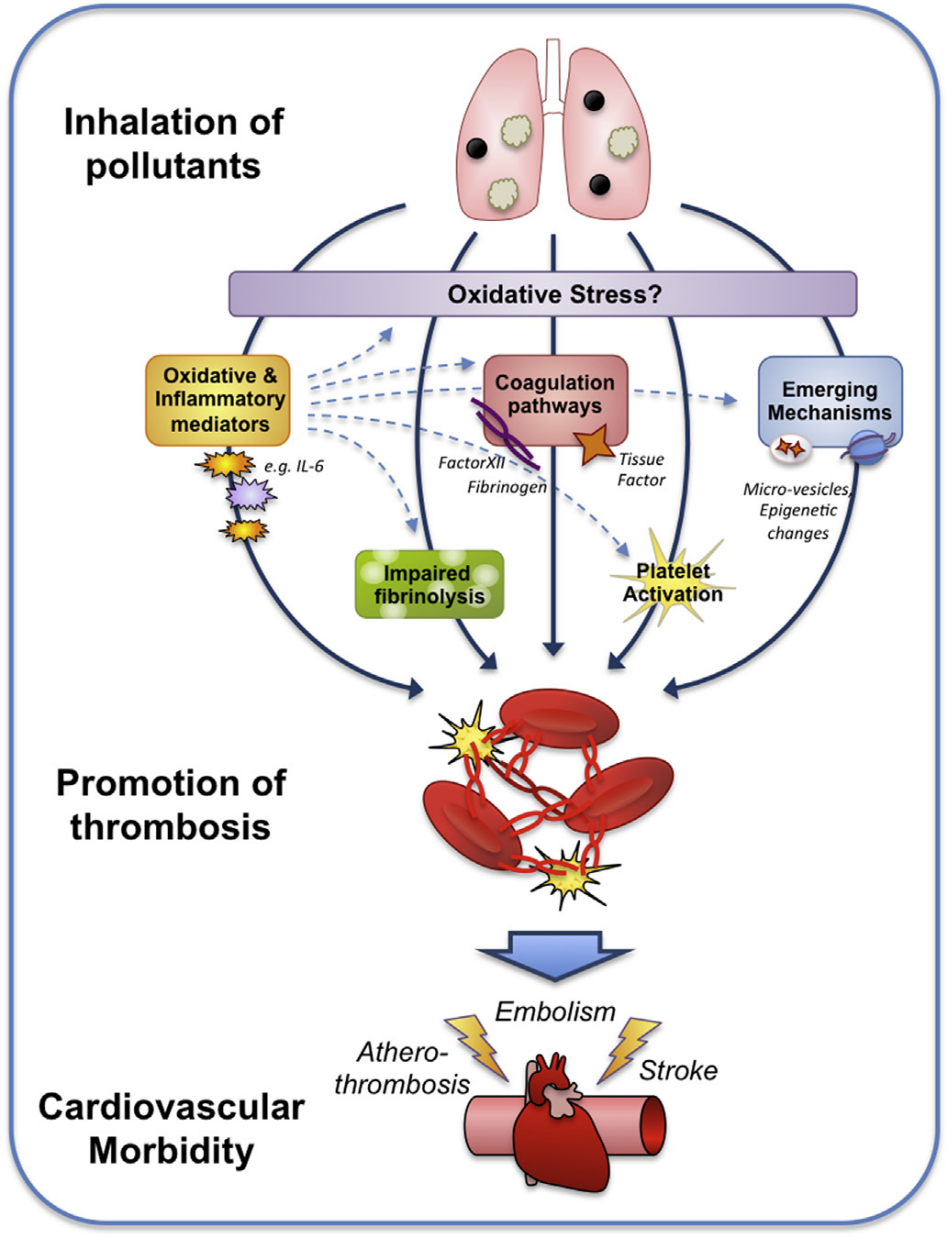
Schematic showing the pathways by which inhalation of pollutants can promote thrombosis increasing the risk of cardiovascular mortality. Oxidative stress may represent a key early pathway that instigates other downstream mechanisms for the pro-thrombotic effect of inhaled pollutants. Additionally, oxidative and inflammatory mediators are likely to interact with these other mechanisms at different stages of their pathways (indicated by dotted lines). Adapted from Robertson & Miller 2018 [222]. Although oxidative stress is specifically indicated as a mechanism in the first pathway, it may play a contributing role in the mechanisms by which the other four pathways exert their actions, and through exacerbation of the resultant cardiovascular pathophysiology these pathways induce.

**Epidemiology**. Thrombotic occlusion of arteries is the predominant cause of heart attacks and strokes. Several epidemiological studies have shown that air pollution, and especially PM exposure, is linked to atherothrombosis, thrombotic stroke and thromboembolism [61]. A plethora of blood markers of pro-thrombotic pathways have been linked to PM exposure, including fibrinogen, tissue factor, von Willebrand factor (vWF), P-selectin and decreases in activity of fibrinolytic pathways the mediate clot breakdown [61,87,222] (Fig. 6). Associations between several air pollutants and P-selectin have been shown to be accompanied by decreases in antioxidant levels in red blood cells [87]. In students in Taipei, Taiwan, both PM_10_ and PM_2.5_ increased in blood 8-OH-dG, alongside increases in fibrinogen, C-reactive protein and the fibrinolytic inhibitor, plasminogen activator inhibitor-1 [80]. In contrast, personal measurement of PM_2.5_ in Copenhagen, Denmark, was associated with greater levels of oxidation of haemoglobin and plasma lipids, although there was no correlation between PM_2.5_ and blood fibrinogen levels [85]. Also, while the use of various assays of oxidative potential of PM was found to have value for predicting pulmonary inflammation, there was only limited value in predicting biomarkers of coagulation [223].

The composition of PM from five locations in the Netherlands has been explored in relation to thrombotic biomarkers [224]. While PM composition did have an influence on biomarker levels, there was a less consistent relationship with the oxidative potential of the PM. A review of human studies concluded that the metallic components of PM are involved in its propensity to induce pro-thrombotic and oxidative biomarkers [225]. Polyunsaturated fatty acids from fish oil have been shown to reduce blood fibrinogen associated with exposure to PM_2.5_ [154].

**Controlled exposures in humans**. Controlled inhalation of dilute DE in healthy volunteers increased blood coagulability in an *ex vivo* model of thrombosis to a damaged arterial wall in physiologically representative conditions [226]. Activation of platelets and platelet-monocyte binding were proposed as the driving mechanism for the increased coagulability, however, the authors speculate a role for oxidative stress and inflammation in the activation of platelets and vascular dysfunction observed in a parallel study with showing increased thrombosis to DE [116]. A separate group of researchers demonstrated that DE alters the expression of several antioxidant pathways in peripheral blood monocytes, supporting a role for systemic oxidative stress in the cardiovascular actions of DE [111]. Another gene profiling study found that inhalation of DE was associated with changes in pathways of both coagulation and oxidative stress/inflammation, including Nrf-2 pathways and inducible forms of NOS [227]. Additionally, controlled exposures to CAPs increased blood plasminogen and markers of acute phase response in individuals with genetic deficiencies in various antioxidant systems [188]. In contrast, a study of young healthy individuals asked to spend 5-h at locations with varying PM levels did not find an association between vWF and the *in vitro* oxidative potential of the different PM [152].

**Animal models**. Pulmonary exposure to PM and DE have also been shown to upregulate vWF and tissue factor, and downregulate fibrinolytic pathways, in rodent models [167]. These changes were associated with alterations in haemoxygenase-1 and LOX-1 pathways. Pulmonary instillation of urban PM [228] or DEP [229] has been shown to accelerate thrombosis at sites of large vessel injury. In contrast, Nemmar and colleagues found an increase in tail bleeding time (potentially indicating a decrease in thrombogenecity) following pulmonary instillation of DEP [230]. The authors speculate that this could be due to decreases in circulating platelet numbers in response to DEP. Nonetheless, a follow-up study found that DEP instillation increased thrombosis in cerebral arterioles after photochemical injury [231]. The changes were accompanied by reduction in antioxidant activity [230,231], and could be reversed by compounds with antioxidant properties [[231], [232], [233]]. The pro-thrombotic effect of DEP in arterioles of a diabetic mouse model were linked to a combined action of increased platelet activation, decreased fibrinolytic activity, systemic inflammation and oxidative stress [234]. Lastly, redox-active transition metals in PM from Mexico City were linked to prothrombotic and anti-fibrinolytic effects in the rat lung [235]. The surface chemistry of the insoluble particulate, rather than the aqueous soluble compounds that can leach from the PM, played a greater contribution to these effects.

***In vitro* experiments**. Direct addition of PM to whole blood has been used to test to the pro-thrombotic potential of particles. The concentrations used in these experiments are likely to be beyond that expected to reach the circulation after inhalation in the real world [236]. Nonetheless, broad insights into the relative potencies of different PM and potential mechanisms may be garnered. Soluble extracts of oil-fly ash containing PM were able to directly promote thrombosis, the extent of which was dependent on metal content of the PM [237]. These effects could be reduced by complexing iron, suggesting that Fenton-derived hydroxyl free radicals could be a potential mechanism. A similar observation has been shown for a US urban PM standard reference material [238]. Free-radical generating capacity of PM was linked to the prothrombotic phenotype of endothelial cells in an *in vitro* model of endothelial-fibrin-clot binding [239]. Direct administration of DEP increased blood coagulation *in vitro*, which could be prevented with the antioxidant/anti-inflammatory agent, emodin [231]. Treatment of cultured endothelial cells with ultrafine PM from Chapel Hill, USA, promoted the ability of these cells to generate active thrombin from a substrate, an effect that was reversed by SOD and catalase, or diphenyleneiodonium (a flavoprotein inhibitor that may act through inhibition of NAD(P)H-oxidase) [240].

### 4.7 Stroke

Stroke is caused by ischaemia in the brain that results in sensorimotor impairments and potentially damage to the brain or dementia. Stroke can be categorised by the cause of the ischaemia; ischaemic stroke caused by atherothrombotic occlusion with the arteries within the brain or those leading to the brain (e.g. the carotid arteries), or haemorrhagic stroke caused by rupture of the cerebral blood vessels and bleeding out into the brain. In either case, ischaemia triggers a cascade of pathophysiological changes in the cells of the affected area, and oxidative stress is a key feature of both the ischaemia and damage caused by sudden reperfusion of the tissues should it occur.

**Fig. 7:**
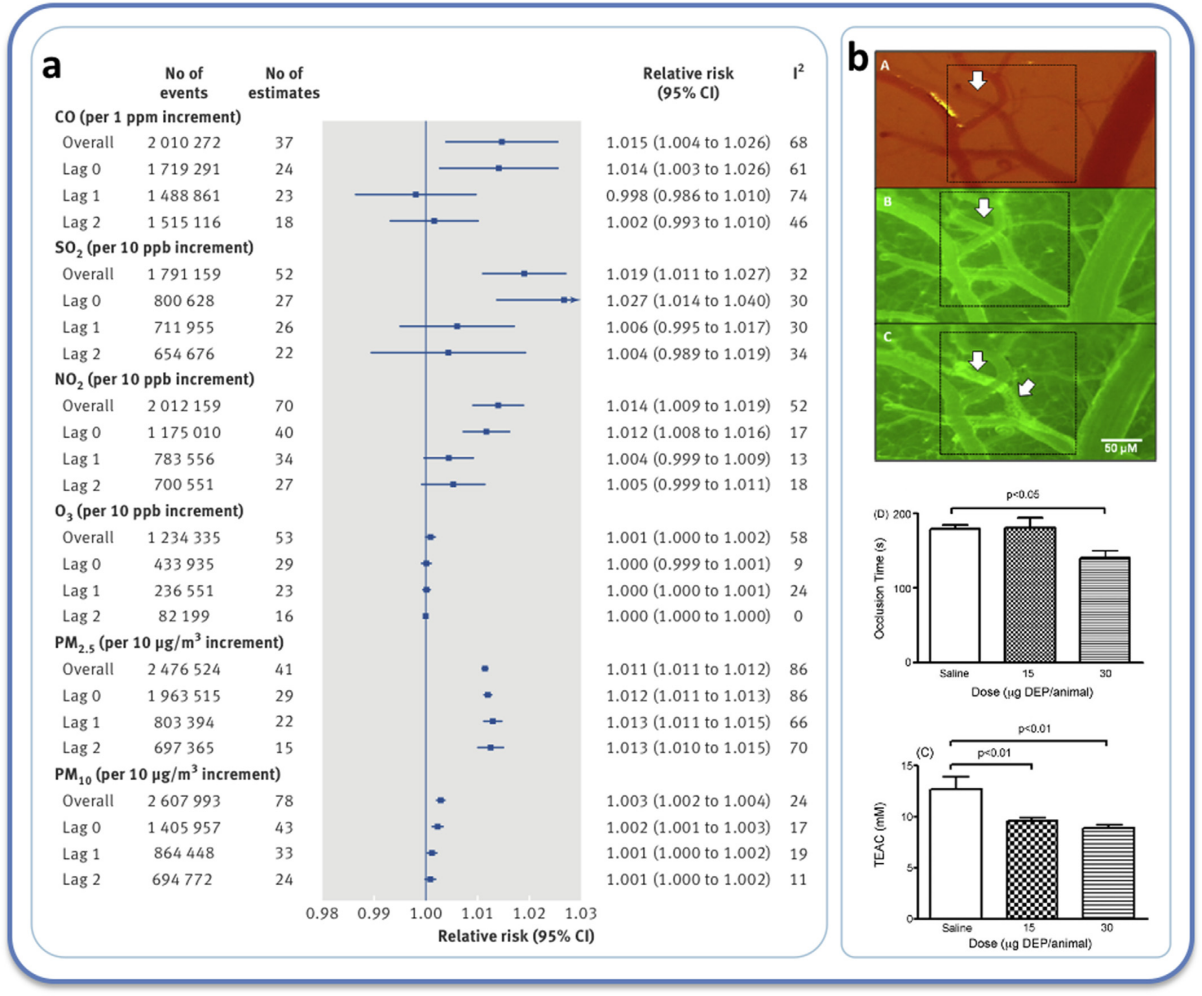
Air pollution exposure is associated with stroke. a) Meta-analysis of the epidemiological evidence that exposure to air pollutants is associated with increase risk of stroke, globally. While the magnitude of the effects for gaseous pollutants can be substantial in some studies, the associations between PM and stroke are more consistent than gaseous co-pollutants. Data from Shah et al. 2015 [54]. b) Thrombotic occlusion of cerebral pial venules in mice following photochemical injury. *Top panel*: accumulation of fluorescein associated with platelets (arrows) to the vessel wall A) prior to injury, B) at time of cessation of blood flow, c) 24-h hours after injury. *Middle panel*: pulmonary administration of diesel exhaust particles (DEP) decreases the time to occlusion, i.e. accelerates the clotting of blood. *Lower panel*: DEP decreased total equivalent antioxidant capacity (TEAC) in the same mice potentially indicating a role of oxidative stress. Data from Nemmar et al. 2009 [232].

**Epidemiology**. There is a wealth of epidemiological evidence showing the exposure to air pollution is associated with increased hospital admission for stroke in different regions of the world [56,57,241]. Associations for PM tend to be more consistent than those for gaseous co-pollutants [54] (Fig. 7) and are observed at levels of air pollution below many guideline levels [242]. However, the role of oxidative stress in these associations has received little attention in epidemiological studies. Although direct evidence is lacking, it has been postulated that free radical generation from PM promotes stroke through increased inflammation and blood coagulability [243], which accords with the stronger associations between air pollution and ischaemic stroke rather than haemorrhagic stroke [241,242]. While not necessarily stroke *per se*, a Swiss study investigated air pollution and oxidative stress in blood samples from non-smoking patients collected within 17 years prior to their diagnosis with cerebrovascular disease [244]. In this study, PM_2.5_ and NO_2_ were all associated with methylation of genes linked to oxidative stress and inflammation.

**Animal models**. While it is challenging to fully reproduce the causes and consequences of stroke, *in vivo* and *in vitro* models have provided evidence to suggest that air pollution would exacerbate stroke, with oxidative stress being an anticipated mechanism [56]. Roadside ultrafine PM, with a known ability to generate free radicals *in vitro*, was shown to alter glutamate signalling and cytokine release in the brain following inhalation in rats [245]. Upregulation of haemoxygenase expression was observed in the brain after instillation of PM_10_ in mice [246]. The effects of PM on the brain have been associated with levels of transition metals in PM, potentially suggesting a role for metal-derived free radical generation [247,248]. Oil-combustion PM was shown to cause marginally greater changes in oxidative stress markers in the lungs and heart of stroke-prone hypertensive rats, in comparison to either healthy or hypertensive rats that are not prone to stroke [249]. Oxidative stress was proposed to play a contributing role in disruption of the blood brain barrier after inhalation of vehicle exhausts [134,250,251]. Indeed, photochemical induction of thrombosis in cerebral microvessels was exacerbated by pulmonary exposure to DEP; an effect that could be prevented by co-administration of an antioxidant cysteine pro-drug [232].

***In vitro* experiments**. Cells within the central nervous system are considered to have a lower reserve of antioxidants in comparison to many other cell types [252]. Cultured neuronal cells and microglial (the macrophage-like cell of the nervous system) have been used to show that PM can induce oxidative stress and cytotoxicity [56]. Free radical generation from roadside PM regulated N-methyl-d-aspartate (NMDA) channels (implicated in hypoxic neuronal damage) in cultured neuronal and glial cells [245]. Similar observations have been found for the direct action of DEP on dopamine signalling in PC-12 cells (used as a model for neural differentiation) [253]. Additionally, periods of ischaemia can be induced in cultured brain slices leading to molecular changes that have representative features of the response to cerebral ischaemic *in vivo*. Application of these tissue preparations with various types of PM can induce cellular-molecular changes characteristic of the response to stroke (e.g. alterations in NMDA channel activity, dopaminergic and glutamatergic disruption). In many cases, such effects are associated with inflammation and oxidative stress (see Ref. [56]).

Primary cultures of microvascular cells can be used to model cerebral arteries. Generation of reactive oxygen species are a prominent mechanism by which DEP can impair the function of endothelial cells that line the inner surface of such arteries [160,254]. These cells represent a potential early target in the disease processes underlying cerebrovascular disease. Direct exposure of DEP to isolated brain capillaries increased oxidative stress and inflammation, and may alter blood brain barrier permeability [134]. While there is debate as to whether inhaled particles can access the brain in sufficient numbers, and penetrate throughout cerebral tissue, these *in vitro* findings offer a means by which inhalation of (the smallest ultrafine) particles could confer increased susceptibility to the consequences of a stroke.

## 5 Conclusions

Awareness of health effects of air pollution is increasing globally, and has now become a priority issue on the environmental, health and political agenda. This awareness has been bolstered by global data revealing the staggering magnitude of the health effects of pollution; culminating in annual mortality rates in excess of several million people. Additionally, over the last three decades, a formidable body of scientific evidence on the underlying biology has grown to support a case for causality in the health effects of air pollution. A crucial stimulus for action has been the wider recognition that the effects of air pollution are not confined to the lung, but instead have effects in what appears to be every organ of the body. The cardiovascular effects of pollution are increasingly gaining mainstream recognition. Given the high incidence of cardiovascular disease worldwide, and the high mortality rates that accompany it, the effects of pollution on the cardiovascular system will remain of key importance. Furthermore, now there is robust human evidence that inhaled nanoparticles may enter the bloodstream [23], the circulation is not only a means to deliver translocated particles throughout the body, but also the direct action of pollutants on the cardiovascular function could contribute to the effects of air pollution on different organs of the body (e.g. through hypertension, impaired perfusion of organs, alterations in vascular growth, etc.) [5].

**Fig. 8:**
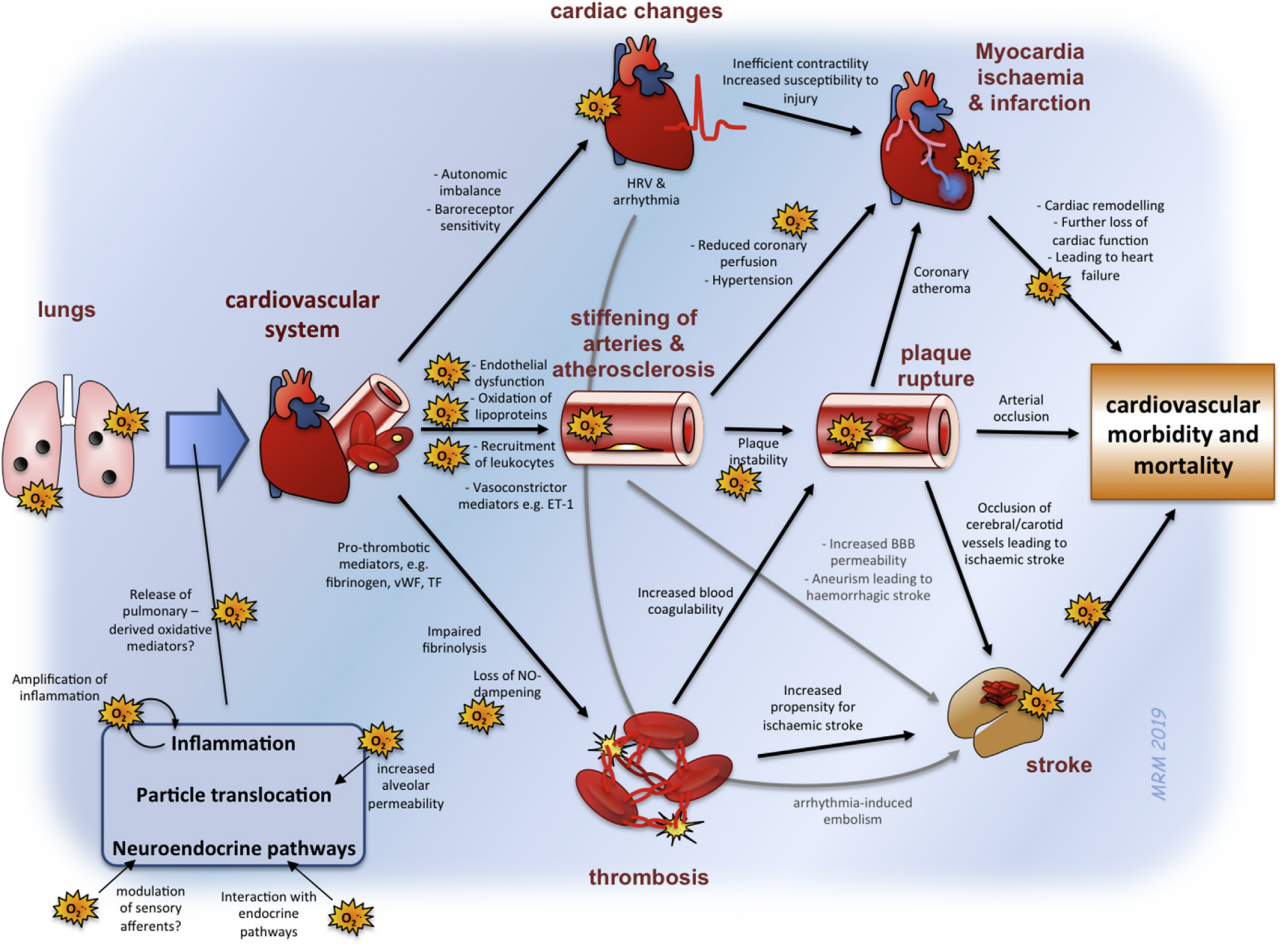
Contribution of oxidative stress to the mechanisms by which inhaled PM induces cardiovascular dysfunction. A complex series of interconnecting mechanisms underlies the effects of inhaled PM on cardiovascular morbidity and mortality. O_2_^-.^ is used to represent places where oxidative stress is likely to play a direct role in exacerbating the disease process. While a direct action of oxidative stress is not always immediately apparent, it is worth noting that oxidative stress has been associated with most of the cardiovascular impairments shown on the diagram, and may indirectly contribute to other pathways by which PM has cardiovascular actions. Abbreviations: BBB = blood brain barrier; ET-1 = endothelin-1; HRV = heart rate variability; NO = nitric oxide; TF = tissue factor; vWF = von Willebrand Factor.

Substantial progress has been made in determining the biological mechanisms for the cardiovascular effects of air pollution. A host of interacting mechanisms has been revealed, however, oxidative stress represents a key mechanism for the pathophysiological actions of pollution on the different facets of the cardiovascular system (Fig. 8). It is notable that oxidative stress emerges as a mechanism in the cardiovascular actions of pollutants using different research approaches; across epidemiological studies, controlled exposures in man, animal models and *in vitro* preparations. Clearly there are inconsistencies in evidence and, even before publication bias, studies will inevitably favour positive results over negative findings (and it should be acknowledged that this is also true to a degree in the present review too). Nonetheless, the sheer scale of the high-quality research, with complementary findings across multiple endpoints and study types, would argue that oxidative stress is a crucial mechanism underlying the link between air pollution and cardiovascular disease. Whether or not oxidative stress is the key initiating event, or only a contributing factor, is challenging to address, however, its presence will undoubtedly exacerbate disease. The close interplay between oxidative stress and inflammation represents a likely means by which the actions of air pollution can be amplified to produce pathophysiological effects in multiple organs. Furthermore, given the clear pro-oxidative effects of many pollutants, and capacity for oxidative stress to impair multiple aspects of cardiovascular function, oxidative stress is likely to play a key mediating role, as opposed to simply being an epiphenomenon of the later stages of disease.

Reducing of the sources of pollution should be the key strategy to alleviate the burden of air pollution on health. However, given the challenges faced in lowering anthropogenic pollution in the face of increasing urbanisation, and the inevitably sluggish progress in implementing political and lifestyle changes, there is a place for interventions that can protect against pollution in the intervening time. Medicines should not be a first-line strategy to prevent the effects of pollution, however, this does not mean that such interventions should be dismissed entirely, especially for those that may be at greater risk (e.g. the young, elderly, those with pre-existing cardiorespiratory disease, and those that have an unavoidable high exposure to pollutants). The consistency of the evidence for a role of oxidative stress in the actions of air pollution raises the potential for the use of antioxidant compounds to ameliorate the actions of exposure to pollutants. This is especially the case given that dietary changes can be a simple means to increase antioxidant intake. While vitamin supplements have largely fallen out of favour given the disappointing large-scale trials with cardiovascular endpoints, it should not be forgotten that these supplements are readily-available, low cost and largely innocuous, and there is substantial scientific evidence supporting their potential to lessen aspects of disease. Indeed, this review highlights several preclinical studies [7,8,255] (see also work by Nemmar et al. e.g. Ref. [256]), as well as a few small panel studies [[257], [258], [259]] whereby antioxidants can prevent the cardiovascular effects of air pollution. Of note, a recent study of over half a million subjects in the USA demonstrated that a Mediterranean diet rich in antioxidants (higher proportion of fruit and vegetables, olive oil, oily fish and moderate consumption of alcohol (e.g. antioxidant-rich red wine)) led to a reduction in the rates of cardiovascular mortality (from ischaemic heart disease, cardiac arrest and stroke) associated with PM_2.5_ and NO_2_ exposure [259]. Polyunsaturated fatty acids from fish oil have also been shown to increase antioxidant enzyme levels and decrease peroxidised lipid/lipoproteins in the blood [154,187,260,261], as well as ameliorate the effects of PM_2.5_ on HRV [186,187], blood fibrinogen, plasma inflammatory markers and endothelin-1 [154]. Similar positive effects of fish oils have been found for endothelial dysfunction caused by controlled exposure to PM_2.5_ [114]. Further large scale studies in human volunteers are needed to ascertain if this approach has merit as an adjunct to strategies to reduce pollutants.

In conclusion, oxidative stress is a key mechanism by which exposure to air pollution causes cardiovascular morbidity and mortality. Strategies that reduce air pollutants would be expected to reduce the burden of cardiovascular disease, and potentially other associated health conditions driven by oxidative pathways.

## Declaration of competing interest

The author has no conflicts of interest to declare.
